# Efficacy and safety of lenalidomide in the treatment of B-cell non-Hodgkin lymphoma

**DOI:** 10.1007/s12672-024-00965-7

**Published:** 2024-04-05

**Authors:** Yang Liu, Yanju Li, Chike Zhang, Xu Yang, Bo Yang, Jinyang Cheng, Juan Chen, Xiaoshuang Yuan, Ya Li, Ying Chen, Fengqi Zhang, Dongxin Tang, Zhixu He, Feiqing Wang

**Affiliations:** 1 Clinical Medical Research Center, The First Affiliated Hospital of Guizhou University of Traditional Chinese Medicine, No. 71 Bao Shan North Road, Yunyan District, Guiyang, 550001 Guizhou China; 2 Department of Hematology Oncology, Affiliated Hospital of Guizhou Medical University, No. 28 Guiyi Street, Yunyan District, Guiyang, 550004 Guizhou China; 3 Key Laboratory of Adult Stem Cell Translational Research, Chinese Academy of Medical Sciences, Guizhou Medical University, Guiyang, Guizhou China; 4 Academy of Medical Engineering and Translational Medicine, Tianjin University, Tianjin City, China

**Keywords:** Mantle cell lymphoma, Follicular lymphoma, Marginal zone lymphoma, Diffuse large B cell lymphoma, Lenalidomide

## Abstract

**Background:**

The combination of rituximab and chemotherapy is a first-line treatment for patients with B-cell non-Hodgkin lymphoma. Lenalidomide is an immunomodulatory drug that has shown promising properties and activity in a variety of hematological malignancies. This study evaluated the efficacy and safety of lenalidomide-based regimens in the treatment of B-cell non-Hodgkin lymphoma.

**Methods:**

The PubMed, Science Direct, ClinicalTrials.gov, and Web of Science databases were searched for relevant studies published up to May 2022. Studies with patients diagnosed with non-Hodgkin B-cell lymphoma, who were randomly assigned to a lenalidomide treatment group or a non-lenalidomide control group were considered for inclusion in this review and meta-analysis. Pooled hazard ratios (HRs) with 95% confidence intervals (CIs) of the time-to-event outcomes and risk ratios (RRs) with 95% CIs of dichotomous data were estimated.

**Results:**

A total of 3593 patients from 10 studies were evaluated. The results of the pooled analysis indicated that the lenalidomide-based regimen was associated with prolonged overall survival (HR, 0.85; 95% CI 0.74–0.97; *P* = 0.02) and progression-free survival (HR, 0.70; 95% CI 0.57–0.88; *P* = 0.002). Significant differences were found in the overall response rate (RR, 1.18; 95% CI 1.04–1.33; *P* = 0.01) and complete response rate (RR, 1.18; 95% CI 1.00–1.39; *P* = 0.05) between the treatment and control groups.

**Conclusions:**

Lenalidomide appears to be a promising therapeutic agent that offers the possibility of a novel combination of chemotherapy free regimen for patients with B-cell non-Hodgkin lymphoma.

**Supplementary Information:**

The online version contains supplementary material available at 10.1007/s12672-024-00965-7.

## Introduction

Non-Hodgkin lymphoma is one of the most common cancers, with its incidence ranking eighth and tenth among men and women, respectively. Countries with a high human development index have higher incidence rates than countries with a low human development index [1]. The majority non-Hodgkin lymphomas are B-cell derived, B-cell non-Hodgkin lymphoma originates from B-lymphocytes at different stages of development [2–4]. The B-cell non-Hodgkin lymphomas include malignancies with different morphological, biological, and clinical presentations, and B-cell non-Hodgkin lymphoma is divided into aggressive and indolent lymphomas [5]. Among them, the diffuse large B-cell and the follicular lymphomas are the most common aggressive and indolent lymphomas, respectively [6]. Diffuse large B-cell lymphoma and follicular lymphoma, together, account for 60% of non-Hodgkin lymphomas [7, 8]. The use of rituximab has revolutionized the treatment of B-cell non-Hodgkin lymphoma by targeting specific B-cell surface molecules to remove tumor cells, thereby improving patient survival significantly [9–11]. Although the outcomes have improved in most patients with standard chemoimmunotherapy, patients with refractory or relapsed B-cell non-Hodgkin lymphoma have a poor prognosis [12–14]. The side effects and progressive drug resistance associated with cytotoxic chemotherapy are well-known [15, 16], and the development of chemotherapy-related cardiovascular diseases is relatively common due to the high cardiotoxicity of doxorubicin and cyclophosphamide currently used in first-line treatment regimens for B-cell non-Hodgkin lymphoma [17, 18].

The advent of novel targeted agents and cellular immunotherapy has increased the tendency to use chemotherapy-free regimens with a high safety level, durable response, and lower risk of disease progression, as a substitute for current chemo-immunotherapy [19, 20]. The oral immunomodulatory drug, lenalidomide, has been investigated and used in a variety of hematological malignancies [21]. Lenalidomide exerts anti-tumor effects through direct anti-tumor activity and has effects on the tumor micro-environment through multiple mechanisms; it also has a synergistic action with rituximab in the treatment of B-cell non-Hodgkin lymphoma [22]. The synergistic mechanism of this combination therapy promotes the complementary effects of lenalidomide in combination with other monoclonal agents and small-molecule inhibitors [23]. Lenalidomide has also shown effective activity in patients with relapsed/refractory B-cell non-Hodgkin lymphoma [24].

A better understanding of the efficacy and adverse effects of lenalidomide are likely to influence the choice of treatment options for B-cell non-Hodgkin lymphoma. The existing research on the topic is mostly based on retrospective studies and phase I/II clinical trials on the role of lenalidomide in diffuse large B-cell lymphomas. However, there are no study of treatment with lenalidomide in B-cell non-Hodgkin lymphoma based on large scale clinical trials [25]. Therefore, we conducted a study to examine the efficacy of lenalidomide in the treatment of B-cell non-Hodgkin lymphoma to determine whether treatment with lenalidomide versus no lenalidomide reduces the risk of adverse effects.

## Methods

This study were conducted in accordance with the Preferred Reporting Items of Systematic Reviews and Meta-Analysis (PRISMA) reporting guidelines [26].

### Eligibility criteria

Published studies that met the following eligibility criteria were included in the review and meta-analysis: (1) all patients were diagnosed with non-Hodgkin B-cell lymphoma; (2) lenalidomide-based treatment regimens were used; (3) non-lenalidomide-based treatment regimens were used as a control; (4) the primary outcomes were overall survival and progression-free survival, and the secondary outcomes were the overall response rate, the complete response rate, and safety; and (5) the study’s design was either a randomized controlled trial (RCT) or a retrospective cohort study. We did not impose any restrictions on publication status, date of publication, or language of the studies. Multiple reports from the same study were considered to be one publication, and only articles with recent and complete data were included. Full-text articles and supplementary appendices for trials were used as resources. Reviews, editorials, and conference papers without sufficient data were excluded.

### Information sources and search strategy

Online databases, including PubMed, Science Direct, ClinicalTrials.gov, and Web of Science were searched from inception to May 2022 to identify eligible studies. The following MeSH terms and relevant variants in English were used as search terms: “Lymphoma, B-Cell”; “Lymphoma, Follicular”; “Lymphoma, Mantle-Cell”; and “Lenalidomide.” References that were included in the relevant studies were manually searched. Two reviewers independently screened the titles and abstracts of the articles, excluded unrelated articles, and then selected the articles that conformed to the study’s criteria for inclusion by browsing through the full text.

### Data extraction and quality assessment

Data from the selected studies were extracted using a standardized form, which included the surname of the first author, year of publication, location of the study, ClinicalTrials.gov Identifier, trial phase, histology, experimental protocol, sample size, mean age, follow-up time, and outcome events. Hazard ratios and 95% confidence intervals (CIs) of patients’ overall survival and progression-free survival were extracted from survival curves and survival data. The number of events, the corresponding population’s overall response, the complete response, and adverse events were extracted. If a concurrent independent review was available, the data assessed by the independent review committee were extracted.

The risk of bias among the RCTs was evaluated based on the following items: generation of an allocation sequence, allocation concealment, blinding, incomplete outcome reporting, selective outcome reporting, and other biases. Each item of the trial was graded as low, unclear, or high risk for bias according to the criteria specified in the Cochrane Handbook. The Newcastle–Ottawa Scale was used to assess three aspects (with eight questions each) to determine the quality of the retrospective studies: selection, comparability, and results. Data extraction and quality assessment were evaluated independently by two authors, and all disagreements were resolved through discussion.

### Statistical analysis

We calculated the pooled hazard ratios (HRs) of the time-to-event outcomes using the inverse variance and reported the pooled estimates and corresponding 95% confidence intervals (CIs). Risk ratios (RR) and 95% CIs of the dichotomous data were calculated using the Mantel–Haenszel method. Heterogeneity among the studies was assessed using the I^2^ statistic. I^2^ values of 25, 50, and 75%, respectively, were considered to be low, medium, and high levels of heterogeneity. We analyzed the data using a fixed-effects model, and we used a random-effects model when I^2^ > 50%. Assessments of quality and risk of bias were performed for all studies. We conducted subgroup analyses of overall and progression-free survival by type of lymphoid neoplasm. Differences between the subgroups were assessed using the Chi-square test. Sensitivity analysis was performed for all results, especially for results with high heterogeneity. For outcomes with a specific number of patients who were lost to follow-up, we assumed that the outcomes were most severe among those patients. Results were considered statistically significant when the *P*-value was ≤ 0.05. All statistical analyses were completed using Review Manager, version 5.3.

## Results

### Study selection and quality assessment

**Fig. 1:**
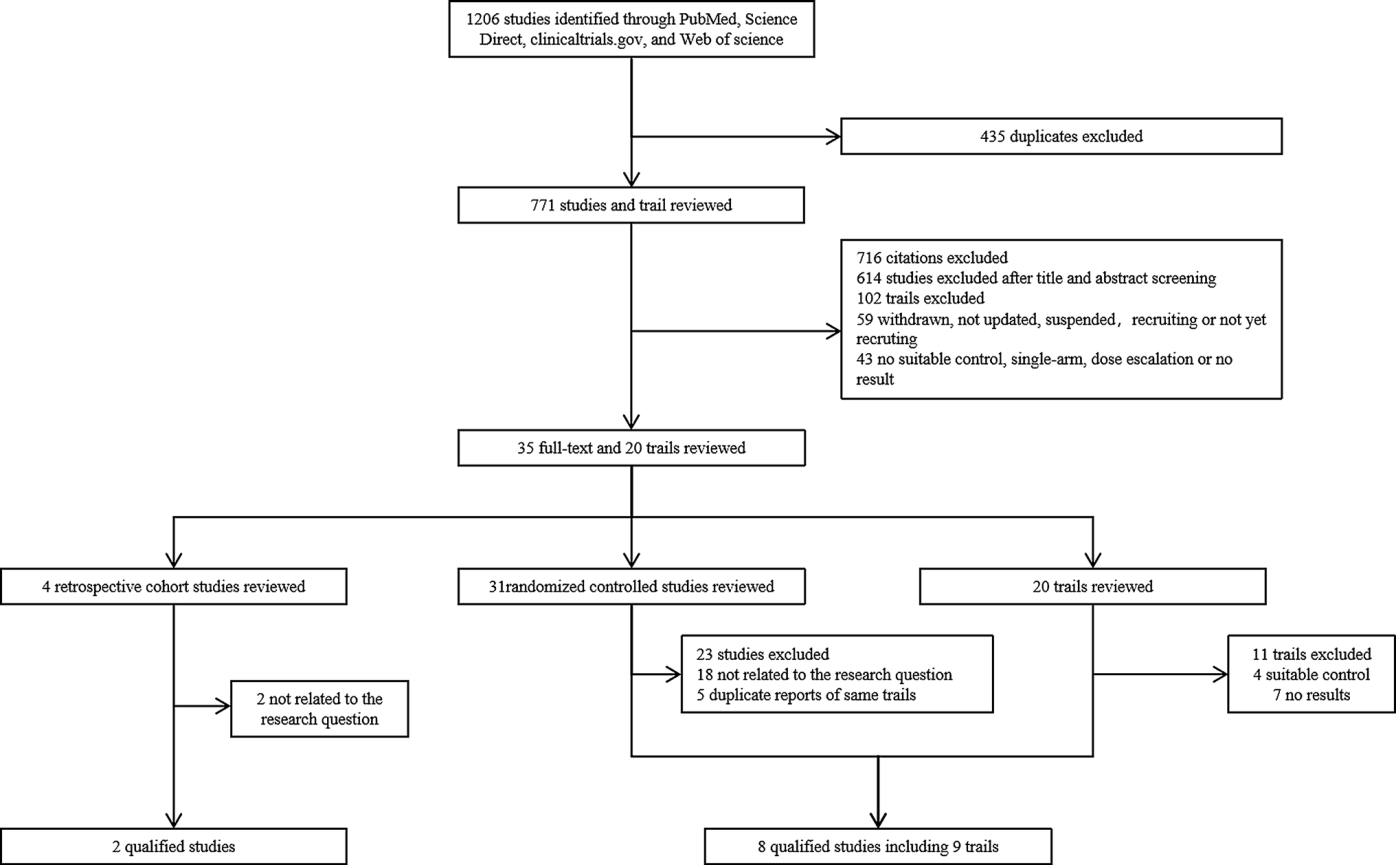
Flow diagram of the selection of studies

A total of 1206 titles and abstracts in the initial literature search were identified, and 771 citations were retained after we removed all duplicates (Fig. 1). After assessing the titles and abstracts of all the included articles to determine compliance with the inclusion criteria, 614 articles were excluded because they were irrelevant to the studies' research questions and 102 clinical trials were excluded because they were irrelevant to the research questions or they had insufficient data. The full text of the remaining 35 articles were reviewed, and 25 studies were excluded, including 20 articles with unanswered but relevant research questions and five articles with reports of the same experiment in different periods. Nine of the 20 experiments matched the screening criteria, and another 11 articles were excluded because of the absence of appropriate controls and lack of adequate data. No new studies were found after reviewing the relevant literature and the reference lists from relevant studies for the present review. Finally, 10 published studies that met the established inclusion criteria were included in this systematic review [27–36].

### Study characteristics

Characteristics of the individual studies are described in Table 1. A total of 3593 patients were included in the present study. These 10 selected studies included eight prospective studies and two retrospective cohort studies. One experiment included patients with mantle cell lymphoma, four experiments included patients with follicular lymphoma and marginal zone lymphoma, and five included patients with diffuse large B-cell lymphoma. The median follow-ups ranged from 4.9 to 96 months. Except for one study with a target population over 80 years old, the median ages of the included patients ranged from 59 to 73.6 years. A higher incidence of neutropenia, thrombocytopenia, leukopenia, anemia, and febrile neutropenia was found among patients with grade 3 or more severe hematological toxicity. A higher incidence of fatigue, diarrhea, infection, and rash was found in patients with grade 3 or more severe non-hematological toxicity.

**Table 1:** Characteristics of the included trials

Author year	Sources	Location countries	Trial no	Phase	Population	Regimen	N	Median age, years	Median follow up, months
Cheah et al. [27]	Annals of oncology	United States	/	/	FL	R2	94	NA	40.8
						R-CHOP	119	NA	96
Zucca et al. [28]	Blood	Norway, Sweden, Switzerland	NCT01307605	II	FL	R^2^	77	61	48
						R	77	63	48
Ayers et al. [29]	Clinical Lymphoma, Myeloma & Leukemia	United States	/	/	R/R DLBCL	L	83	73.6	22.7
						B/G	300	73.1	22.7
Morschhauser et al. [30]	New England Journal of Medicine	Japan, United States, Australia, Belgium, Canada, France, Germany, Italy, Portugal, Spain	NCT01476787 NCT01650701	III	FL	R^2^	513	59	37 .9
						R-CHOP/R-CVP/RB	517	59	37 .9
Nowakowski et al. [31]	Journal of Clinical Oncology	United States	NCT01856192	II	DLBCL	R^2^-CHOP	145	67	36
						R-CHOP	135	66	36
Nowakowski et al. [32]	Journal of Clinical Oncology	Australia, Belgium, Canada, China, Czechia, France, Ireland, Israel, Italy, Japan, Korea, Netherlands, New Zealand, Poland, Portugal, Puerto Rico, Russian Federation, Spain, Switzerland, Turkey, United States	NCT02285062	III	ABC-DLBCL	R^2^-CHOP	285	65	27.1
						Placebo Plus R-CHOP	285	65	27.1
Leonard et al. [33]	Journal of Clinical Oncology	Belgium, Brazil, China, Czechia, France, Germany, Israel, Italy, Japan, Poland, Portugal, Puerto Rico, Russian Federation, Spain, Turkey, United Kingdom, United States	NCT01938001	III	R/R FL R/R MZL	R^2^	178	64	28.3
						Placebo Plus R	180	62	28.3
Oberic et al. [34]	Journal of Clinical Oncology	Belgium, France	NCT02128061	III	DLBCL	R^2^-CHOP	122	> 80	25.1
						R-CHOP	127	> 80	25.1
Trněný et al. [35]	Lancet Oncology	Belgium, Czechia, Denmark, France, Germany, Greece, Israel, Italy, Netherlands, Poland, Russian Federation, Spain, Sweden, United Kingdom	NCT00875667	II	R/R MCL	L	170	68.5	15.9
						IC	84	68.5	15.3
Czuczman et al. [36]	Clinical Cancer Research	Australia, Austria, Czechia, France, Italy, Spain, Sweden, United Kingdom, United States	NCT01197560	II/III	R/R DLBCL	L	51	69	6.8
						IC	51	65	4.9

OS	PFS	ORR(%)	CR(%)	PR(%)	SD(%)	PD(%)	≥ Grade 3 hematological toxicity. n (%)	≥ Grade 3 non-hematological toxicity. n (%)
3-year = 97%	3-year = 87%	NA	NA	NA	NA	NA	NA	NA
3-year = 92%	3-year = 60%	NA	NA	NA	NA	NA	NA	NA
4-year = 91%	Median = 5.0 years	77.9	61.0	16.9	2.6	1.3	Neutropenia 23.4% Thrombocytopenia 3.9% Anemia 1.3%	Fatigue 2.6% rash 5.2% Infections 3.9% Urinary tract infection 2.6%
4-year = 90%	Median = 2.3 years	57.1	36.4	20.8	9.1	3.9	Neutropenia 5.3% Febrile neutropenia 1.3%	Fatigue 1.3% rash 1.3% Infections 2.6%
Median = 15.4 months	NA	NA	NA	NA	NA	NA	NA	NA
Median = 7.7 months	NA	NA	NA	NA	NA	NA	NA	NA
3-year = 94%	3-year = 77%	60.8	48.1	12.7	0.4	17.0	Neutropenia 31.6% Thrombocytopenia 2.2% Leucopenia 1.6% Febrile neutropenia 2.2%	Diarrhea 2.0% Rash 3.9% Vomiting 0.4%
3-year = 94%	3-year = 78%	65.0	53.0	12.0	0	15.3	Neutropenia 50.1% Thrombocytopenia 1.6% Leucopenia 6.0% Febrile neutropenia 6.6%	Diarrhea 1.2% Fatigue 0.8% Nausea 1.6% Vomiting 1.4%
3-year = 83%	3-year = 73%	97	73	NA	NA	NA	Neutropenia 36.1% Thrombocytopenia 20.5% Anemia 17.5% Febrile neutropenia 15.1%	Lung infection 4.2% Sepsis 4.2% Fatigue 6.0% Hypokalemia 3.6%
3-year = 75%	3-year = 62%	92	68	NA	NA	NA	Neutropenia 31.6% Thrombocytopenia 7.6% Anemia 11.7% Febrile neutropenia 8.2%	Lung infection 2.9% Sepsis 3.5% Fatigue 3.5% Hypokalemia 1.2%
2-year = 79%	2-year = 67%	90.9	69.1	21.8	2.1	1.8	Neutropenia 59.7% Thrombocytopenia 16.6% Leukopenia 14.1% Anemia 22.2% Febrile neutropenia 13.7%	Diarrhea 2.1% Fatigue 1.8%
2-year = 80%	2-year = 64%	90.9	64.9	26.0	0.7	1.4	Neutropenia 48.2% Thrombocytopenia 11.3% Leukopenia 14.8% Anemia 13.7% Febrile neutropenia 8.8%	Pyrexia 3.5% Peripheral sensory neuropathy 1.8% Diarrhea 1.1% Fatigue 1.1%
2-year = 93%	Median = 39.4 months	77.5	33.7	43.9	11.2	3.9	Neutropenia 50.0% Thrombocytopenia 2.3% Leukopenia 6.8% Anemia 4.5%	Diarrhea 2.8% Upper respiratory tract infection 1.1% Infusion-related reaction 2.2%
2-year = 87%	Median = 14.1 months	53.3	18.3	35.0	30.6	12.8	Neutropenia 12.8% Thrombocytopenia 1.1% Leukopenia 1.6% Anemia 0.5%	Upper respiratory tract infection 2.2% Pyrexia 1.6%
2-year = 65.7%	2-year = 54.8%	82	58	NA	NA	NA	Neutropenia 32.5% Thrombocytopenia 7.7% Anemia 9.4%	Infections and infestations 14.5% GI disorders 5.1%
2-year = 66.0%	2-year = 56.2%	73	53	NA	NA	NA	Neutropenia 17.7% Thrombocytopenia 0.8% Anemia 5.6%	Infections and infestations 9.7% GI disorders 6.5%
Median = 27.9 months	Median = 8.7 months	40.0	4.7	35.3	29.4	20	Neutropenia 43.7% Thrombocytopenia 18.0% Leucopenia 7.8% Anemia 8.4%	Diarrhoea 3.6% Pyrexia 2.4% Pneumonia 3.6%
Median = 21.2 months	Median = 5.2 months	10.7	0	10.7	52.4	31.0	Neutropenia 33.7% Thrombocytopenia 27.7% Leucopenia 10.8% Anemia 7.2%	Pyrexia 1.2% Pneumonia 2.4%
Median = 7.8 months	Median = 3.4 months	27.5	9.8	17.6	25.5	47.1	Neutropenia 42.6% Thrombocytopenia 16.7% Leukopenia 3.7% Anemia 18.5% Febrile neutropenia 7.4%	Infections and infestations 18.5% Gastrointestinal disorders 16.7% Metabolism and nutrition disorders 16.7%
Median = 6.2 months	Median = 2.0 months	11.8	2.0	9.8	21.6	64.7	Neutropenia 25.5% Thrombocytopenia 25.5% Leukopenia 12.7% Anemia 30.9% Febrile neutropenia 3.6%	Infections and infestations 25.5% Gastrointestinal disorders 10.9% Metabolism and nutrition disorders 20.0%

*NA* not available, *OS* overall survival, *PFS* progression-free survival, *ORR* overall response rate, *CR* complete response, *PR* partial response, *SD* Stable disease, *PD* progressive disease, *N* Number of patients enrolled, *FL* follicular lymphoma, *DLBCL* diffuse large B-cell lymphoma, *MCL* mantle cell lymphoma, *MZL* marginal zone lymphoma, *R/R* relapsed and/or refractory, *L* lenalidomide, *R* rituximab, *R*^*2*^ lenalidomide + rituximab, *CHOP* cyclophosphamide + doxorubicin + vincristine + prednisone, *CVP* cyclophosphamide + vincristine + prednisone, *B* bendamustine, *G* gemcitabine, *IC* investigator’s choice

Eight of the studies were multi-center RCTs, six of which were open-label trials. Two trials explicitly used blinding, with one using masking for the participants, care providers, investigators, and outcome evaluators. The study populations of the two retrospective studies were representative samples, and their study designs were between-group comparisons. However, no missing data were reported in the retrospective studies and all the included studies were judged to be of high quality.

### Overall survival

**Fig. 2:**
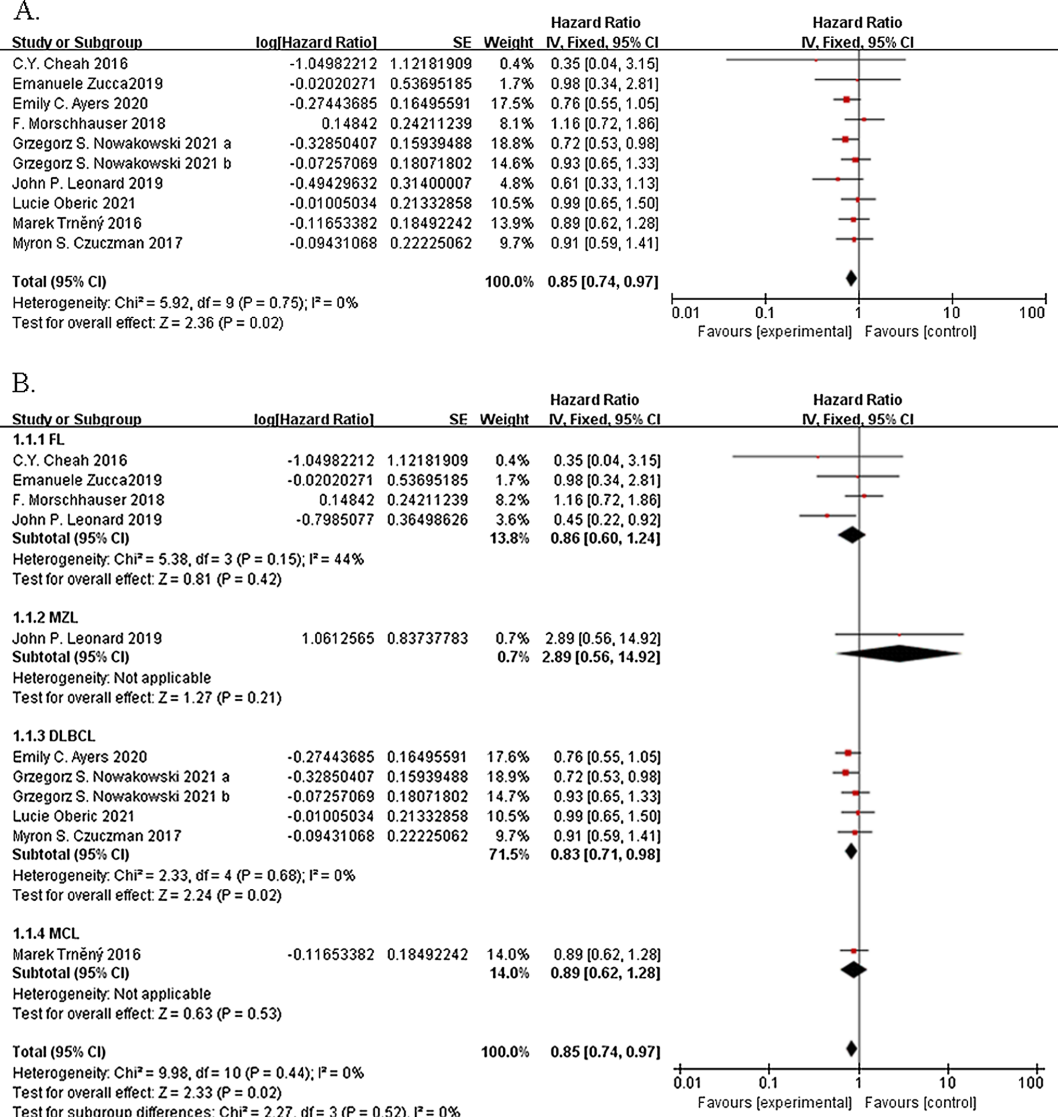
Forest plots of the hazard ratios of the overall survival of the lenalidomide group versus the no-lenalidomide group. **A** All trials are included. **B** The subgroup analysis of overall survival was divided by type of lymphoid neoplasm

The pooled analysis of overall survival was consistent among the 10 included studies. A significant improvement was found in the overall survival of patients receiving lenalidomide (HR, 0.85; 95% CI 0.74–0.97; *P* = 0.02), compared to those treated without lenalidomide (Fig. 2A). No significant heterogeneity (I^2^ = 0%) was observed between the experiments, and the funnel plots did not support publication bias (Additional file 1: Fig. S1). A subgroup analysis of overall survival showed that the patients with diffuse large B-cell lymphoma clearly benefitted from their treatment with lenalidomide (HR, 0.83; 95% CI 0.71–0.98; *P* = 0.02; I^2^ = 0%; 5 trials; 1,584 patients; Fig. 2B). However no significant difference was found between the treatment and control groups with follicular lymphoma (HR, 0.86; 95% CI 0.60–1.24; *P* = 0.42; I^2^ = 44%; 4 trials; 1692 patients), marginal zone lymphoma (HR, 2.89; 95% CI 0.56–14.92; *P* = 0.21; 1 trial; 63 patients) or mantle cell lymphoma (HR, 0.89; 95% CI 0.62–1.28; *P* = 0.53; 1 trial; 254 patients).

### Progression-free survival

**Fig. 3:**
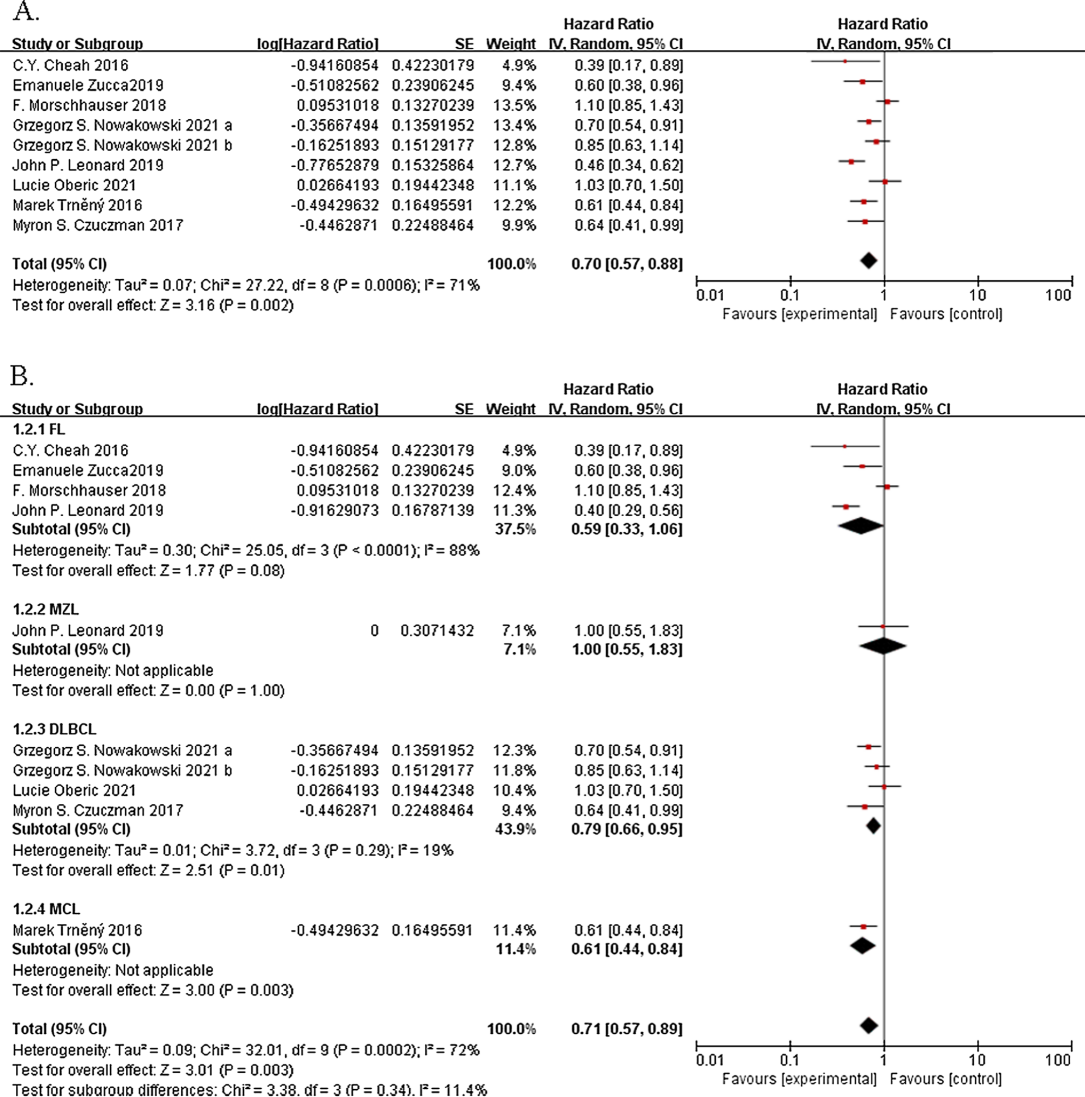
Forest plots of the hazard ratios of the progression-free survival of the lenalidomide group versus the no-lenalidomide group. **A** All trials are included. **B** The subgroup analysis of progression-free survival was divided by type of lymphoid neoplasm

In the pooled analysis of progression-free survival, the lenalidomide-based treatment was associated with a significantly prolonged progression-free survival, compared to the non-lenalidomide-based treatments. The pooled HR of the nine studies showed a significant improvement in progression-free survival (HR, 0.70; 95% CI 0.57–0.88; *P* = 0.002; I^2^ = 71%; Fig. 3A). Furthermore, progression-free survival was analyzed by lymphoid neoplasm (Fig. 3B). In the four experiments including 1692 patients with follicular lymphoma, the addition of the lenalidomide group probably contributed little or no difference to their progressive-free survival (HR, 0.59; 95% CI 0.33–1.06; *P* = 0.08; I^2^ = 88%). An experiment with 63 relapsed/refractory marginal zone patients with lymphoma showed no changes between treatments (HR, 1.00; 95% CI 0.55–1.83; *P* = 1.00). A trial including 254 patients with relapsed/refractory mantle cell lymphoma revealed that the lenalidomide arms of the study had significantly higher progression-free survival than the non-lenalidomide arms had (HR, 0.61; 95% CI 0.44–0.84; *P* = 0.003). Patients with diffuse large B-cell lymphomas were enrolled in five studies, and four studies that included 1201 patients reported progressive-free survival. Lenalidomide significantly improved the progressive-free survival of patients with diffuse large B-cell lymphoma (HR, 0.79; 95% CI 0.66–0.95; *P* = 0.01; I^2^ = 19%), but no statistically significant differentce was found between the subgroups. However, those patients with the activated B-cell subset (known as the ABC Type) of diffuse large B-cell lymphoma who received lenalidomide treatment showed a significant improvement in progressive-free survival (HR, 0.75; 95% CI 0.59–0.95; *P* = 0.02; I^2^ = 19%; Additional file 1: Fig. S2).

### Overall and complete response rates

**Fig. 4:**
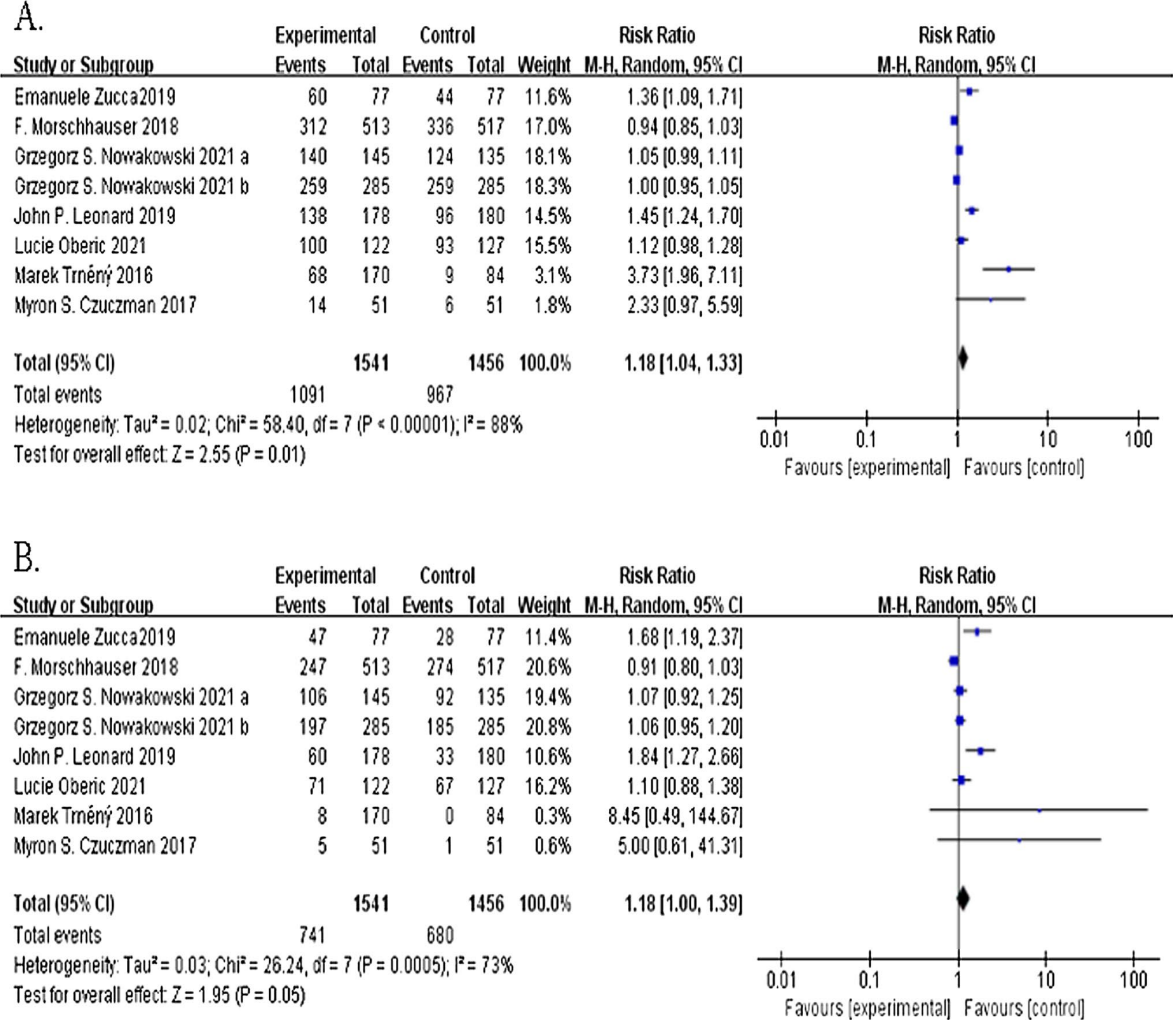
Forest plots of the risk ratios of the overall response and complete response of the lenalidomide group versus the no-lenalidomide group. **A** Overall response. **B** Complete response

Eight trials including 2,998 patients reported an overall response rate. A total of 1,091 of the 1,541 patients in the lenalidomide group responded to treatment, and 967 of the 1,456 patients in the control group responded to treatment. The addition of lenalidomide significantly improved the overall response rate (RR, 1.18; 95% CI 1.04–1.33;* P* = 0.01; I^2^ = 88%; Fig. 4A), and compared to the control group, the lenalidomide group showed a significantly improved complete response rate (RR, 1.18; 95% CI 1.00–1.39; *P* = 0.05; I^2^ = 73%; Fig. 4B).

### Adverse events

An incidence of grade 3 or more severe adverse events was reported in 1,360 patients in three trials, and no evidence was found that the lenalidomide-based treatment increased the incidence of adverse events above grade 3 (RR, 1.11; Table 2). Among the patients with hematological toxicity, lenalidomide-based treatment was associated with an increased risk of tertiary and more severe neutropenia (RR, 1.55; 8 trials; 3023 patients) and anemia (RR, 1.45; 7 trials; 2013 patients), compared with the controls. No significant difference in the risk of tertiary or more severe thrombocytopenia (RR, 1.44; 8 trials; 3023 patients) or leukocytopenia (RR, 0.73; 5 trials; 2292 patients) was found between the treatment groups. Among the patients with non-hematological toxicity, the risk of diarrhea (RR, 2.56; 5 trials; 2336 patients) and rash (RR, 8.64; 3 trials; 1519 patients) increased in the lenalidomide group. However, the risk of nausea was reduced significantly in the lenalidomide group (RR, 0.29; 3 trials; 1933 patients), whereas the risk of fatigue (RR, 1.29; 7 trials; 2782 patients) was not significantly different between the groups. Finally, the pooled analysis of the risk ratio of second primary cancers was not statistically significant between the patients treated with the lenalidomide-based protocol and the controls (RR, 0.88; 5 trials; 2010 patients).

**Table 2:** Pooled analysis of risk ratios of adverse events above grade 3 and second primary cancers

Events	Experimental group	Control group	RR	95% CI	P	I^2^, %
	n/N (%)	n/N (%)				
Adverse events	467/678 (68.9)	457/682 (67.0)	1.11	0.86–1.42	0.43	87
Neutropenia	629/1547 (40.7)	534/1476 (36.2)	1.55	1.03–2.33	0.03	93
Thrombocytopia	147/1547 (9.5)	93/1476 (6.3)	1.44	0.85–2.45	0.18	70
Anemia	136/1040 (13.1)	90/973 (9.2)	1.45	1.13–1.85	0.003	40
Leucocytopenia	75/1187 (6.3)	91/1105 (8.2)	0.73	0.34–1.57	0.42	76
Diarrhea	28/1210 (2.3)	9/1126 (0.8)	2.56	1.25–5.23	0.01	0
Rash	26/760 (3.4)	3/759 (0.4)	8.64	2.62–28.49	0.0004	22
Fatigue	24/1430 (1.7)	18/1352 (1.3)	1.29	0.71–2.34	0.41	0
Nausea	3/966 (0.3)	13/967 (1.3)	0.29	0.09–0.86	0.03	29
Second primary cancers	70/1044 (6.7)	74/966 (7.7)	0.88	0.64–1.21	0.45	0

*n/N* number (n) with outcome/number (N) in treatment group, *RR* risk ratio, *95% CI* 95% confidence interval, *P* P value

## Discussion

This study was conducted using 10 studies to assess the safety and efficacy of a lenalidomide-based treatment regimen in patients with B-cell non-Hodgkin lymphoma. The results of the included studies showed that the progressive-free survival and overall survival of patients treated with lenalidomide improved, compared to patients who were not treated with lenalidomide. The subgroup analyses of the overall and progressive-free survival were performed considering possible differences in efficacy among patients with different B-cell non-Hodgkin lymphomas. The results showed that diffuse large B-cell lymphoma and mantle cell lymphoma responded well to it, and follicular lymphoma and marginal zone lymphoma achieved efficacy similar to that expected after standard treatment. Moreover, they also had increased overall and complete response rates. Although lenalidomide increased the risk of grade 3 neutropenia, anemia, diarrhea, and rashes to a higher (more severe) grade among the patients receiving it, no significant difference in the risk of thrombocytopenia, leukopenia, or fatigue was found. To date, this comprehensive assessment is the first and largest study of lenalidomide-based treatment for B-cell non-Hodgkin lymphoma, and it provides the highest level of evidence for both physicians and patients.

The main result of the present study was the significant difference in overall survival between the lenalidomide and no-lenalidomide groups. There are two possible explanations for lenalidomide's advantage: its direct anti-tumor activity and its synergistic effect with rituximab. The efficacy of lenalidomide may vary by lymphoma cytogenetics and tumor microenvironment. In order to identify which patients will receive the maximum benefit from treatment containing lenalidomide, we performed a subgroup analysis to explore which patients with B-cell non-Hodgkin lymphoma of different lymphoma types might benefit from treatment with lenalidomide. The results of our analysis showed that lenalidomide-based treatment improved overall survival in patients with diffuse large B-cell lymphoma. Nevertheless, among patients with follicular, marginal zone, and mantle cell lymphomas, lenalidomide-based treatment was associated with similar long-term survival benefits of standard treatment, and it demonstrated meaningful clinical activity, which is consistent with the results reported in previous studies [37, 38]. A non-chemotherapeutic regimen containing lenalidomide may be more appropriate for older patients who are difficult to treat with standard immuno-chemotherapy. Although the data are insufficient to support the subgroup analysis, studies show that the activity of lenalidomide is maintained in patients aged > 65 years old. Furthermore, long-term follow-up results of experiments are needed because they may demonstrate the effect of lenalidomide on overall survival more accurately, especially experiments including indolent lymphoma.

Given that the overall survival of patients with B-cell non-Hodgkin lymphoma was prolonged significantly after the introduction of rituximab into treatment and that overall survival is influenced by many factors. We focused on progressive-free survival, compared to prolonged overall survival. Our analysis showed that the addition of lenalidomide was associated with longer progression-free survival. Among these were significant differences in diffuse large B-cell lymphoma and mantle cell lymphoma. Moreover, the poor prognosis of activated B-cell (ABC) diffuse large B-cell lymphoma also significantly improved progression-free survival, consistent with previous study articles and pre-clinical studies of lenalidomide in diffuse large B-cell lymphoma [39]. In two previous studies, a long-term follow-up trial of lenalidomide combined with rituximab for follicular lymphoma, which had an 8 year progression-free survival of 65%, challenged the clinical trial outcome of bendamustine plus rituximab with a 5 year progression-free survival of 65.5% [40]. Hence, the natural course of follicular lymphoma (the most common indolent lymphoma) might determine the need for long-term follow-up to achieve meaningful progression-free survival.

Patients less than 60 years old with diffuse large B-cell lymphoma who were treated with complete and partial remission 24 months after their diagnosis had a survival rate comparable to age- and sex-matched healthy individuals [41]. The patients with B-cell non-Hodgkin lymphoma who relapsed after 5 years had better survival than those with earlier relapses [42]. The median follow-up of the response evaluations for most of the included experiments was longer than 24 months. Our study showed that patients receiving lenalidomide had an 18% higher likelihood of response and complete response, compared with controls. Moreover, at the end of treatment for aggressive lymphoma and follicular lymphoma, the use of positron emission tomography and computed tomography is the standard test of complete remission [43]. However, some trials did not use positron emission tomography as a means of assessment, and the complete response rate may have been underestimated [44].

Given the significant improvements in overall survival and progression-free survival among B-cell lymphoma patients, the management and prevention of treatment-related side effects have received increasing attention. A summary analysis of adverse events showed that adding lenalidomide did not increase the risk of grade 3 or more severe adverse events, compared to the control groups. The adverse events most associated with the use of lenalidomide were neutropenia, rashes, and diarrhea, which were the result of loss of key transcription factors inducing granulocyopoiesis and the activation of dendritic-mediated cell humoral immunity [45]. The most common cases of hematotoxicity were neutropenia, thrombocytopenia, anemia, and leukopenia. The results showed an increased risk of grade 3 or more severe neutropenia and anemia, with no significant difference in thrombocytopenia or leukopenia. However, previous studies have shown that neutropenia during lenalidomide treatment is reversible and improved by sustained growth factor support [43]. Although the risk of a rash is increased and may hinder treatment due to its negative effects on quality of life, the incidence of rashes is not significant. A grade 1 or 2 rash can be monitored or treated with steroids, and a rash above grade 3 can be controlled by interrupted treatment and prednisone [46]. With prolonged survival, a second primary cancer with a clear effect on survival became more prevalent in patients with B-cell non-Hodgkin lymphoma [47]. Non-Hodgkin lymphoma has a greater risk of a second tumor, which does not significantly differ between subtypes [48]. The occurrence of a second tumor was associated with immune dysfunction [49, 50]. There was no evidence in the summary analysis that patients treated with lenalidomide might be prone to a second primary cancer. Lenalidomide was also found to be associated with venous thromboembolic events in the treatment of B-cell non-Hodgkin lymphoma [51]. However, given the few reports of thromboembolic events in the included literature, no relevant results were reported in the present study.

The strengths of this study include a comprehensive search that was based on clear inclusion criteria. Most of the included studies were multicenter RCTs conducted at prestigious research centers, with large samples of representative populations. The data from the prospective RCTs in the present study may be more robust than the data from previous reviews, which were mainly based on cohort studies and single-arm clinical trials, thereby providing more reliable conclusions and clinical guidance. Moreover, the evidence from the prospective RCTs and retrospective studies was carefully evaluated and the risk of bias for all of them was low. The overall survival of patients with diffuse large B-cell lymphoma found in our studies was largely consistent with that of previous studies [25], so we performed a more detailed analysis of progressive-free survival, overall survival, and adverse reactions. Our results provide answers to efficacy and safety questions about lenalidomide in the treatment of B-cell lymphoma. A better understanding of lenalidomide's mechanism of action on cells in the tumor micro-environment will help optimize the therapeutic effect of lenalidomide on B-cell non-Hodgkin lymphoma.

This study has several limitations. First, the dosage and mode of administration of lenalidomide and the drugs included in the combination treatment varied among the different studies. Therefore, a standard lenalidomide dose or treatment regimen could not be recommended based on this study. The short follow-up period was insufficient, and some of the experiments never reached the overall or progressive-free survival medians. The included literature contained different subtypes of B-cell non-Hodgkin lymphoma, with histological inconsistencies. Although our results provide answers to efficacy and safety questions about lenalidomide in the treatment of B-cell lymphoma, they also highlight gaps in the research. Analyses of RCTs on mantle cell, marginal zone, and other B-cell non-Hodgkin lymphomas that were not included in the present study’s analysis, are needed to obtain a clearer understanding of the efficacy and safety of lenalidomide. Given the ability of lenalidomide and its simplified treatment as an oral drug, the dose and course of treatment should be determined, and patient satisfaction and quality of life, should be investigated. A better understanding of lenalidomide's mechanism of action on cells in the tumor micro-environment will help optimize the therapeutic effect of lenalidomide on B-cell non-Hodgkin lymphoma.

## Conclusions

This study sheds light on the addition of lenalidomide to the treatment regimen, which may be associated with significant survival benefits and controlled adverse effects. Based on the findings of existing clinical trials, this study provides a relatively high level of evidence for the efficacy and safety of lenalidomide in the treatment of B-cell non-Hodgkin lymphoma.

### Supplementary Information

**Additional file 1: Fig S1.** Funnel plot to assess for publication bias. **Fig S2.** Forest plot of hazard ratios of progression free survival in ABC-Type diffuse large B cell lymphoma.

## Data Availability

The datasets used and analysed during the current study are available from the corresponding author on reasonable request.

## Abbreviations

95% CI: 95% Confidence interval
ABC DLBCL: Activated B-cell-like diffuse large B-cell lymphoma
B: Bendamustine
CHOP: Cyclophosphamide + doxorubicin + vincristine + prednisone
CR: Complete response
CVP: Cyclophosphamide vincristine prednisone
DLBCL: Diffuse large B cell lymphoma
FL: Follicular lymphoma
G: Gemcitabine
HR: Hazard ratio
IC: Investigator’ s choice
L: Lenalidomide
MCL: Mantle cell lymphoma
MZL: Marginal zone lymphoma
n/N: Number (n) with outcome/number (N) in treatment group
N: Number of patient enrolled
NA: Not available
ORR: Overall response rate
OS: Overall survival
PD: Progressive disease
PFS: Progression free survival
PR: Partial response
R: Rituximab
R^2^: Lenalidomide + rituximab
RR: Risk ratio
R/R: Relapsed and/or refractory
SD: Stable disease
