# Supplementary material for: Efficacy and safety of lenalidomide in the treatment of B-cell non-Hodgkin lymphoma
Source: Discov Oncol. 2024 Apr 5;15:105. doi: 10.1007/s12672-024-00965-7 (PMC10997569; doi:10.1007/s12672-024-00965-7)
Supplement: Supplementary file 1 — Additional file 1: Fig S1. Funnel plot to assess for publication bias. Fig S2. Forest plot of hazard ratios of progression free survival in ABC-Type diffuse large B cell lymphoma. [file 12672_2024_965_MOESM1_ESM.docx]

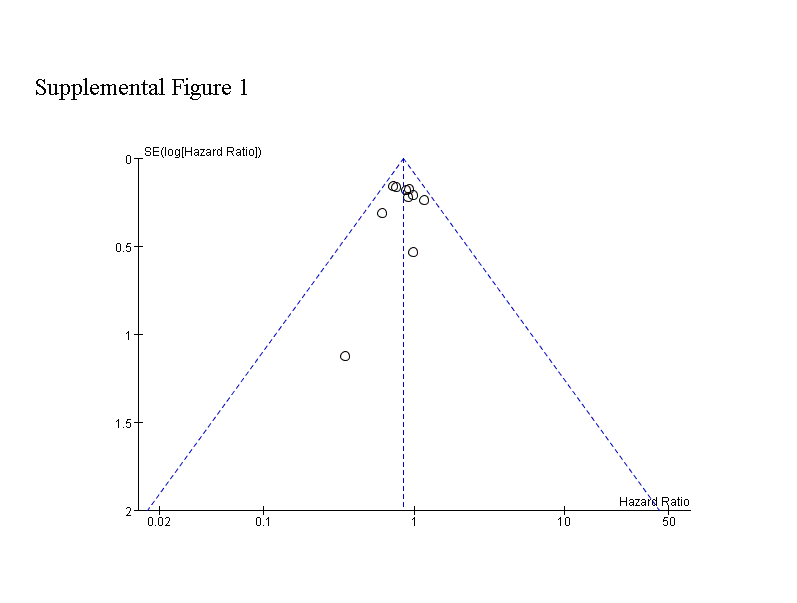


Additional file 1: Fig. S1: Funnel plot to assess for publication bias


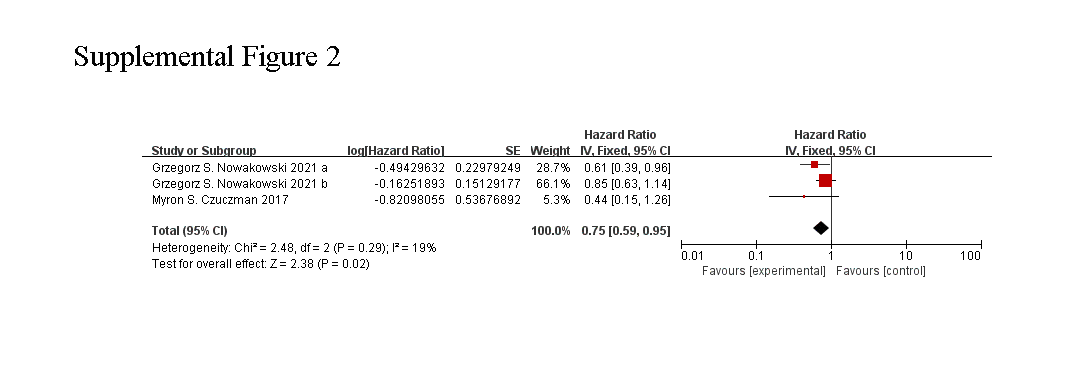


Additional file 1: Fig. S2: Forest plot of hazard ratios of progression free survival in ABC-Type diffuse large B cell lymphoma.
